# Prevalence of uncontrolled hypertension and its associated factors in 50–74 years old Iranian adults: a population-based study

**DOI:** 10.1186/s12872-023-03357-x

**Published:** 2023-06-24

**Authors:** Fariba Farhadi, Roqayeh Aliyari, Hossein Ebrahimi, Hassan Hashemi, Mohammad Hassan Emamian, Akbar Fotouhi

**Affiliations:** 1 Student Research Committee, School of Nursing & Midwifery, Shahroud University of Medical Sciences, Shahroud, Iran; 2 Department of Epidemiology, School of Public Health, Shahroud University of Medical Sciences, Shahroud, Iran; 3 Center for Health Related Social and Behavioral Sciences Research, Shahroud University of Medical Sciences, Shahroud, Iran; 4 Noor Research Center for Ophthalmic Epidemiology, Noor Eye Hospital, Tehran, Iran; 5 Ophthalmic Epidemiology Research Center, Shahroud University of Medical Sciences, Shahroud, Iran; 6 Department of Epidemiology and Biostatistics, School of Public Health, Tehran University of Medical Sciences, Tehran, Iran

**Keywords:** Population-Based Study, Prevalence, Related Factors, Uncontrolled Hypertension

## Abstract

**Background:**

By the lengthening of life span, the incidence of chronic diseases such as hypertension and uncontrolled hypertension has increased. This study aims to determine the prevalence of uncontrolled hypertension and its related factors in the age group of 50–74 years in Shahroud, northeast Iran.

**Methods:**

The data of the third phase of the Shahroud Eye Cohort Study were used in this study. This phase of the cohort study included 4394 participants aged 50 to 74 years from the previous phases. In addition to ophthalmological and optometric examinations, demographic characteristics, blood biochemistry tests, and blood pressure measurements were performed in this phase. Individuals with a blood pressure ≥ 140/90 mm/Hg (being treated or not treated with antihypertensive medicines) were defined as uncontrolled hypertension. In patients with diabetes and chronic kidney disease, blood pressure equal to or higher than 130/80 mm/Hg was considered uncontrolled hypertension. Descriptive statistics and multiple logistic regression were used to analyze the data.

**Findings:**

Overall, the prevalence of uncontrolled hypertension out of all the participants was 61.7% (95% CI: 60.3–63.2). Multiple regression results showed that the male gender (OR: 2.1, 95% CI: 1.5–2.9), patients with diabetes (OR:3.2, 95% CI: 2.4–4.3), and patients with chronic kidney disease (CKD) (OR: 3.2, 95% CI: 2.5–4.1) increased the risk of uncontrolled hypertension while in patients with cardiovascular disease (OR: 0.6, 95% CI: 0.4–0.8) and polypharmacy (OR: 0.2, 95% CI: 0.1–0.2) reduced the risk of uncontrolled hypertension.

**Conclusion:**

The present study showed that uncontrolled hypertension has a high prevalence, and factors such as male gender, diabetes, and CKD are associated with this disorder. So, it is recommended to take the necessary measures to formulate and implement immediate actions to prevent or control hypertension.

## Introduction

Nowadays, aging is considered a universal phenomenon. The increase in life expectancy and the decrease in fertility in the world have caused the elderly to have the promptest population growth among various age groups [1]. According to the latest population forecasts, the “life expectancy” at birth in the world has risen from 64.2 to 1990 to 72.6 in 2019 and is expected to exceed 77.1 in 2050 [1]. Moreover, one in every 11 individuals in the world was 65 years or older in 2019 (9%), and it is predicted that by 2050, it will be one in every six individuals (16%) [1]. Between 2000 and 2030, the worldwide population of individuals older than 65 will increase from 6.9 to 12% worldwide and from 15.5 to 24.3% in Europe. Currently, Asia has the highest rate of elderly in the world, a situation that is projected to continue for at least the next 50 years [2]. During around 42 years, the elderly population in Iran has increased from 5% to 1976 to 10% in 2019 (doubling of older adults), and again, during about 21 years, in 2041, it will reach 20% (re-doubling of the elderly population); However, in many developed countries, the elderly population has doubled during over more than one hundred years, and Iran is one of the countries with the highest acceleration of the older adults in the world [3].

Hypertension is a serious medical condition that significantly increases the risk of cardiac, brain, and kidney diseases. The prevalence of hypertension varies in different regions and income levels. According to the WHO, Africa (27%) and the Americas (18%) have the highest and lowest prevalence of hypertension, respectively [4]. One of the global goals for NCD is to reduce the prevalence of hypertension by 33% between 2010 and 2030 [4]. Hypertension is the leading cause of premature death worldwide. It is estimated that 1.28 billion adults aged 30 to 79 worldwide suffer from high blood pressure, most of whom (two-thirds) live in low- and middle-income countries [4]. Uncontrolled hypertension is a major public health challenge among patients with hypertension, both in high-income and low-income countries [5–7]. It is estimated that 46% of adults with hypertension are unaware of their disease. Also, less than half of adults (42%) with hypertension are diagnosed and treated. In addition, high blood pressure is controlled in only about 1 in 5 adults (21%) [4]. Evidence has shown that age, gender, nonadherence to a low-salt diet, obesity, smoking, and the number of medications taken were among the factors that increase uncontrolled hypertension [8–10]. The importance of controlling hypertension in the elderly can be understood from the results of this study, which showed that hypertension control strategy interacted with the correlation between frailty and cognitive impairment [11]. Data from a study shows arterial hypertension is associated with cognitive decline and treating the hypertension improve cognitive function in elderly hypertensive patients [12]. Considering the importance of blood sugar control in the elderly, data from a study indicates that, hyperglycemia drives physical impairment in frail and hypertensive older adults independently from diabetes mellitus and HbA1c values [13]. In a systematic review one of the key determinants of hypertension in older adults was overweight/obesity [14]. In addition, another systematic review shows that treating blood pressure to at least < 140/80, or lower if tolerated, confers benefits in cardiovascular outcomes [15].Although hypertension is observed in every age group, 90% of them are diagnosed over the age of 60, a large number of these patients need treatment at a younger age, and 70% do not control their blood pressure properly [16].

In a case-control study, untreated hypertension was associated with a higher risk of stroke than untreated hypertension in 32 countries. Meanwhile, untreated hypertension was also associated with a higher risk of intracerebral hemorrhage than ischemic stroke [17]. In a cross-sectional analysis in six Latin American countries, the prevalence of hypertension was 44%. 53.3% of them were under treatment and controlled blood pressure was reported in 37.6% of the patients under treatment [18]. Also in a previous study, the prevalence of hypertension in 451,755 adults (over 18 years) was 27.9%, and the rates of treatment and control of hypertension were 40.7% and 15.3%, respectively from 2012 to 2015 in China [19].

The prevalence of controlled hypertension in Iran has been reported differently. For example, in a study that used the baseline data of prospective epidemiological research studies of the cohort study in Iran from 2014 to 2020 with 163,770 participants aged 35 to 70 years, the treatment ratios based on JNC7 and ACC/AHA guidelines among adults with hypertension were 82.2% and 50.4%, respectively. Moreover, the controlled blood pressure ratios among patients treated based on JNC7 and ACC/AHA were 75.9% and 46.3%, respectively [20]. In two descriptive studies in Iran, the prevalence of hypertension were23.2% and 37.3%. Also, the prevalence of uncontrolled hypertension were 7.2% and 38.9% [21, 22].

Considering the above-mentioned issues and the differences in reports of the prevalence of controlled and uncontrolled hypertension in various studies in the world and Iran, this study was performed to determine the prevalence of uncontrolled hypertension and related factors in the age group of 50–74 years.

## Methods

**Fig. 1:**
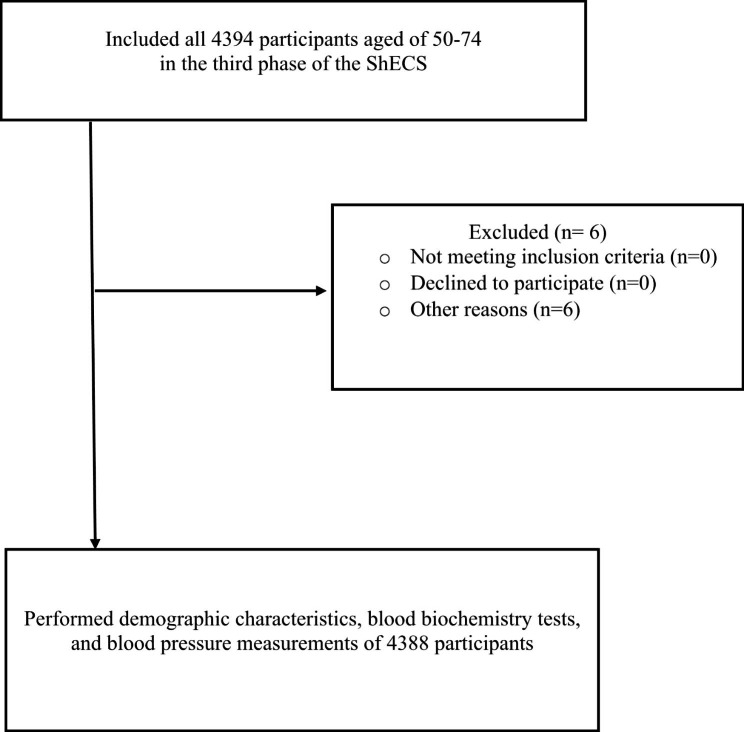
Study flow chart

The present study is part of the Shahroud Eye Cohort Study (ShECS), which was conducted in 2019 and is based on the results of the third phase of that study. ShECS is a longitudinal population-based study. The details of the ShECS methodology have already been provided [23]. The first phase of the study in 2009 included 300 clusters from Shahroud city in 9 strata. At least 20 participants from each cluster aged from 40 to 64 participated in the study. Thus, the total number of participants was 5190 subjects. After explaining the method of the study and obtaining written consent from each participant, one was interviewed, and complete optometry and ophthalmological examination were performed. The demographic factors, employment status, medical history, and ophthalmology were examined during the interview. The second phase of the study was conducted in 2014 with 4737 participants aged 45 to 69. In the third phase, which was conducted in 2019, 4394 people aged 50–74 participated from the participants of the previous phases (85%) of these participants, the information of 4388 people was available in this study (Fig. 1). All middle-aged and elderly participants in the third phase of the ShECS were included in the study, and their blood pressure was measured. The prevalence of uncontrolled hypertension and its related factors were studied (including obesity, age, gender, economic status, dyslipidemia, polypharmacy, diabetes, kidney disease, cardiac disease, smoking, marital status, and education). The participants’ blood pressure was measured by trained nurse staff using a digital and calibrated sphygmomanometer [OMRON (HEM-2228-E): Omron, Kyoto, Japan] from the right arm in a sitting position. A suitable cuff was used (a suitable cuff that covered 80 to 100% of the arm length and approximately two-thirds of the arm length without overlap). Each individual’s blood pressure was measured and recorded twice over 5 min. If the difference between the obtained numbers was more than 10 mm Hg in SBP or 5 mm Hg in DBP, the measurement was performed for the third time. The final blood pressure for each person was the average of the two measurements with the most minor difference [23]. In this study, uncontrolled hypertension was referred to individuals with systolic blood pressure ≥ 140 mmHg or diastolic blood pressure ≥ 90 mmHg and was treated with antihypertensive medicines [24]. Also, the individuals who had systolic blood ≥ 140 mmHg or diastolic blood pressure ≥ 90 mmHg and were not treated, and patients suffering from diabetes and chronic kidney disease (CKD) who had blood pressure ≥ 130/80 mmHg were considered uncontrolled hypertension [25]. The World Health Organization divides the aging period into 45–59 years: middle-aged, 60–74 years: young elderly, 75–90 years: elderly, and 90 years and older: very elderly [26]. The age group of 50–59 who had participated in the study was considered middle-aged, and participants in the 60–74 age group were considered older adults.

The economic status of individuals was calculated by principal component analysis (PCA) based on amenities and home appliances and, accordingly, the individuals were divided into three groups in terms of economic status: high, medium, and low. The weight of each participant was measured using a portable digital scale with an accuracy of 0.1 kg, and their height was measured by an inelastic tape in the standing position without shoes. The Body Mass Index (BMI) was calculated by dividing weight (in kilograms) by height squared (in meters). BMI less and greater than 25 kg/m^2^ were considered normal and overweight or obese, respectively [27].

Participants who had triglycerides above 150 mg/dl (2.26 mmol/lit), cholesterol above 200 mg/dl (6.21 mmol/lit), HDL-C below 40 mg/dl (1.03 mmol/lit), or LDL-Chol above 160 mg/dl were considered the ones with dyslipidemia [28]. Also, those who were diagnosed with dyslipidemia before the interview and were taking lipid-lowering medicines were considered as having dyslipidemia [29]. The participants with fasting blood sugar levels ≥ 126 mg/dl (48 mmol/mol) or the ones with a level of HbA1c ≥ 6.5% were diagnosed as diabetics [23, 30]. Moreover, those who had been diagnosed with diabetes before the interview and were taking blood glucose-lowering drugs were also considered diabetics.

In this study, the cardiac disease was recorded based on the individual’s self-report and that they had been diagnosed with the disease before the interview and were receiving medical treatment.

The Kidney function was calculated by Glomerular Filtration Rate (GFR) according to the following formula [31]:

GFR=(140-age) ×Weight × (0/85if female)/Pcr×72.

The CKD was defined as those with an estimated glomerular filtration rate of less than 60 ml/min per 1.73 m^2^ [32].

Polypharmacy was defined as the concomitant use of several medicines (taking five or more medicines), including OTC medicines or traditional and complementary medicines used by the patient [33].

The data were analyzed by describing the data according to the type of variables using mean and standard deviation for quantitative variables and absolute and relative frequencies for qualitative variables and then the relationship between independent variables with uncontrolled hypertension using multiple logistic regression.

All procedures in the third phase were performed following the ethical standards of the Ethics Committee of Shahroud University under the ethical code of IR.SHMU.REC.1398.039.

## Results

Among the 4394 participants in the third phase of the ShECS, the data required for this study were available for 4388, and the mean age of participants was 61.1 years (53.1% older adults and 46.9% middle-aged). In the present study, the majority of participants (58.8%) were women. The prevalence of uncontrolled hypertension was 61.7% in all participants (95% CI: 60.3–63.2) [60.9% middle-aged (95% CI: 58.7–63) and 62.5% older adult (95%CI: 60.5–64.5)] (Table 1).

**Table 1:** Frequency distribution of blood pressure status in the population of 50–74 years in Shahroud

Blood pressure classification	Number	Prevalence (%)	95% CI*
**Normal**	1076	24.5	(23.2–25.8)
**Controlled**	603	13.7	(12.7–14.8)
**Uncontrolled**	2709	61.7	(60.3–63.2)

*CI: Confidence Interval

According to Table 2 the prevalence of uncontrolled hypertension was 58.2% in women and 66.7% in men. Uncontrolled hypertension was more common in individuals with overweight or obesity, in the elderly, men, middle economic status, individuals with dyslipidemia individuals with polypharmacy, individuals with diabetes, individuals with no CKD, individuals with cardiovascular disease, non-smokers and divorced people. Additional information is listed in Table 2.

**Table 2:** Distributions of patients’ characteristics and clinical aspects of the studied population by blood pressure control

Blood Pressure Classification			Normal			Controlled			Uncontrolled		
Variables			Number (%)	PV ^a^		Number (%)	PV ^b^		Number (%)		PV ^c^
BMI*	**Normal (< 25 kg/m** ^**2**^ **)**		369 (37.0)	< 0.001		111 (11.9)	0.666		478 (51.1)		< 0.001
	**Obese/Overweight (≥ 25 kg/m** ^**2**^ **)**		730 (21.5)			492(14.3)			2228 (64.6)		
**Age Category**	**Middle Age (50-59y)**		585 (28.6)	< 0.001		216 (10.6)	< 0.001		1246 (60.9)		< 0.001
	**Elderly (60-74y)**		486 (21.0)			383 (16.5)			1450 (62.5)		
**Sex**	**Female**		624 (20.2)	< 0.001		410 (16.6)	< 0.001		1443 (58.2)		0.003
	**Male**		397 (23.2)			173 (10.1)			1141 (66.7)		
**Economic Status**	**High**		361 (24.9)	0.086		174 (12.0)	0.025		912 (63.0)		0.061
	**Middle**		324 (22.4)			205 (14.1)			920 (63.5)		
	**Low**		372 (25.7)			221 (15.3)			855 (59.0)		
**Dyslipidemia**	**No**		20 (14.1)	< 0.001		34 (23.9)	0.003		88 (61.9)		0.027
	**Yes**		992 (24.5)			527 (13.0)			2527 (62.4)		
**Polypharmacy**	**Yes**		92 (6.0)	< 0.001		412 (26.9)	< 0.001		1026 (67.1)		< 0.001
	**No**		984 (34.4)			191 (6.7)			1683 (58.9)		
**Diabetes**	**No**		827 (30.2)	< 0.001		372 (13.6)	0.002		1538 (56.2)		< 0.001
	**Yes**		195 (13.0)			200 (13.3)			1109 (73.7)		
CKD*	**No**		334 (17.6)	< 0.001		236 (18.2)	< 0.001		727 (75.7)		< 0.001
	**Yes**		384 (25.7)			145 (6.6)			1654 (56.0)		
**Cardiovascular Disease**	**No**		994 (27.7)	< 0.001		383 (10.7)	< 0.001		2177 (61.2)		< 0.001
	**Yes**		77 (9.3)			220 (26.7)			528 (64.0)		
**Smoking**	**Yes**		27 (20.0)	0.077		26 (19.2)	0.111		82 (60.7)		0.576
	**No**		899 (23.4)			530 (13.8)			2407 (62.7)		
**Marital Status**	**Single**		13 (48.1)	0.044 ^e^		1 (3.7)	0.028 ^e^		13 (48.1)		0.897 ^e^
	**Married**		928 (24.7)			826 (13.2)			2332 (62.0)		
	**Widow**		110 (20.5)			101 (18.9)			323 (60.5)		
	**Divorced**		19 (32.2)			3 (5.1)			37 (62.7)		

a: Comparing between normal blood pressure and controlled HTN; b: Comparing between controlled and uncontrolled HTN, c: Comparing between normal blood pressure and controlled HTN; *BMI: Body Mass Index; *CKD: Chronic Kidney Disease; GFR ≤ 60; e: marital status categorized to married and others

The results of multiple regression on uncontrolled hypertension showed that male gender (OR = 2.1, 95% CI: 1.5–5.9), diabetes (OR = 3.5, 95% CI: 2.4–4.3), and CKD (OR = 3.2, 95% CI: 2.5–4.1) increase the risks of developing uncontrolled hypertension, whereas polypharmacy (OR = 0.2, 95% CI: 0.1–0.2) and cardiovascular disease (OR = 0.6, 95% CI: 0.4–0.8) reduce the risks of developing uncontrolled hypertension. Uncontrolled hypertension was also not associated with obesity or overweight, old age, economic status, dyslipidemia, smoking, and education (Table 3).

**Table 3:** The role of independent variables related to uncontrolled hypertension in the multiple logistic regression model

Independent Variable		Compared to Controlled hypertension	
		Odds Ratio (95% CI*)	P-Value
	Normal (< 25 kg/m^2^)	Reference	
BMI*	Obese/Overweight (≥ 25 kg/m^2^)	1.35 (0.94–1.94)	0.104
**Elderly**		1.04 (0.79–1.38)	0.761
**Sex (Reference: Female)**		2.12 (1.54–2.94)	< 0.001
**Economic Status**	1st Tertile	Reference	
	2nd Tertile	1.18 (0.81–1.73)	0.376
	3rd Tertile	1.15 (0.83–1.60)	0.402
**Dyslipidemia**		1.13 (0.62–2.04)	0.692
**Polypharmacy**		0.20 (0.15–0.27)	< 0.001
**Diabetes**		3.29 (2.47–4.37)	< 0.001
CKD* (Reference: GFR ≤ 60)		0.31 (0.24–0.40)	< 0.001
**Cardiovascular Disease**		0.66 (0.49–0.89)	0.006
**Smoking**		0.69 (0.34–1.41)	0.314
**Education (year)**		0.98 (0.95–1.02)	0.334

*CI: Confidence Interval; *BMI: Body Mass Index; *CKD: Chronic Kidney Disease: GFR ≤ 60

## Discussion

In this study, the prevalence of uncontrolled hypertension was 61.7% (60.9% in the middle-aged and 62.5% in the older adult). The prevalence of uncontrolled hypertension has been reported between 48.6 and 71.8% and 24.1–92.8% in the other countries [32, 34, 35] and Iran [20–22], respectively.

According to a result of a study, overweight and co-morbidity were independent predictors of uncontrolled hypertension. Therefore, early identification and management of co-morbidities among hypertensive patients are crucial for controlling hypertension [36]. Obesity or overweight had no significant relationship with uncontrolled hypertension in this study. The results of a study in the United States showed that although obesity increases the prevalence of hypertension, it has no significant relationship with its control [37]. Also, the results of studies conducted in France, Saudi Arabia, and Africa were inconsistent with the present study [34, 35, 38]. This inconsistency in the findings can be due to the difference in the participant’s age. In the present study, the participants were 50–74 years old, which may have more underlying diseases due to their age. While in the mentioned studies, the participants were over 18 years old.

According to the results of this study, aging had no significant association with uncontrolled hypertension. This finding is consistent with the results of a previous study by Masilela et al. [39]. But, based on the results of a previous study, there was an association between increasing age and the prevalence of hypertension [40]. The Framingham Heart Study [41] showed that more than 90% of the participants with a normal blood pressure [42] at age 55 years eventually develop HTN and approximately 60% of the population has HTN by 60 years of age [41]. The results of a study showed that increasing the stiffness of large arteries causes an increase in vascular resistance in older adults with increased systolic blood pressure [43]. Longitudinal studies showed that in parallel with increasing age, there was a gradual increase in the stiffness of the large elastic artery [44]. The various underlying mechanisms involved in the occurrence of hypertension in older adults have been identified, including changes in mechanical hemodynamics, arterial hardness, neurological and hormonal dysfunction, autonomic dysregulation, and the aging kidney [45]. The vasoconstriction and vascular resistance are responsible for the occurrence of aging in the kidney. This process is due to a decrease in the activity of the calcium adenosine triphosphate and sodium/potassium pumps and a rise in salt sensitivity. In fact, many changes in the arterial vasculature are due to aging [46, 47]. Older adults suffering from uncontrolled hypertension can be attributed to these cases as the main cause of hypertension. But, in previous studies by Almalki et al. (2020), Cherfan et al. (2020) and Aberhe et al. (2020), results showed that ages older than 65 years and also 50 years old were identified as an effective factor in increasing the prevalence of uncontrolled hypertension. This inconsistency in the findings can be due to the difference in the sample size [25, 34, 35].

The results of the present study showed that the prevalence of uncontrolled hypertension was higher in men than women. Also, according to the results of multiple regression analysis, the risk of uncontrolled hypertension was 1.2 times higher in men than women. some studies have found that hypertension is more prevalent among men [48, 49] and also, a global review found a higher mean blood pressure and age-standardized prevalence of hypertension among men [50]. Studies in France, Saudi Arabia, and South Africa have also reported that the male gender is one of the most important risk factors for uncontrolled hypertension, which can be attributed to the fact that men are unhealthier than women [34, 35, 51]. But, in a systematic review which was conducted in Africa, one of the key determinants of systemic hypertension in older adults was the female sex [14].Based on the results of the present study, the economic status of older adults and middle-aged participants had no significant relationship with uncontrolled hypertension. This finding was in line with the results of a study in France [34]. This result can be because most of the participants in this study were covered by health insurance. Despite the difference in economic status, being covered by health insurance has made people likely to face fewer issues in terms of treatment and medication, and the prevalence of uncontrolled hypertension is not different among them.

According to the results, there was no significant relationship between dyslipidemia and uncontrolled hypertension. In line with the results of this study, no significant relationship was observed with dyslipidemia in the Almalki study (2020) [35]. However, Cherfan et al. (2020) showed that dyslipidemia was significantly associated with a higher prevalence and risk of uncontrolled hypertension [34]. Since the patients with dyslipidemia were treated with medication in this study, there was evidence that dyslipidemia has beneficial effects on blood pressure [52], therefore, no significant difference was observed between the two groups. The study by Borghi et al. (2000) reported that patients receiving concomitant antihypertensive and statin therapy experience hypotension. This result cannot be explained simply by the fat-lowering effect of statins or the effects of antihypertensive medicines. These results suggest that using statins in combination with antihypertensive medications may improve blood pressure control in patients with uncontrolled hypertension and high serum cholesterol levels [52].

According to the multiple regression results, the risk of uncontrolled hypertension was 0.20 among patients with polypharmacy compared to others. In a study in France, subjects who took only one antihypertensive medicine had higher uncontrolled hypertension than those who took two or three medications [34]. Polypharmacy indicates multiple comorbidities, each reducing the possibility of controlling blood pressure. Although polypharmacy reduces the chance of uncontrolled hypertension, but because in the current study, the underlying diseases of the people were identified in phases 1 and 2 and they were treated and followed up, it seems that the treatment compliance in people with polypharmacy who had uncontrolled hypertension was more than in people that the number of drugs they used was less than 5 drugs per day.

In this study, the prevalence of uncontrolled hypertension in older adults and middle-aged with diabetes was 73.7%. Also, according to the results, the risk of developing uncontrolled hypertension in people with diabetes was 3.2 times higher than in non-diabetics. In line with this study, Sakboonyarat et al. (2019) showed that diabetes increases the risk of developing uncontrolled hypertension [53]. This effect may be due to insulin resistance and endothelial dysfunction, which causes high blood pressure [54–56]. Co-morbidity has a significant association with uncontrolled hypertension. In the studies in South Asia and China, diabetic and kidney disease co-morbidities were associated with uncontrolled hypertension [10, 42]. Also, a study in Ethiopia showed that co-morbid hypertensive patients were more likely to have uncontrolled hypertension [57]. Many chronic diseases are secondary causes of hypertension so controlling hypertension among hypertensive patients with other chronic co-morbidities might be challengeable. Also, according to a study that conducted in Iran diabetes is one of the predictors of treated high blood pressure [22]. So, this shows more importance of diabetes.

The prevalence of uncontrolled hypertension in older adults and middle-aged with kidney disease was 56%. Moreover, the risk of uncontrolled hypertension in patients with CKD was 3.2 times higher than in non-chronic kidney disease. The results of studies by Almalki et al. (2020) and Gebremichael et al. (2019) were in line with the results of this study [35, 36]. CKD causes an increase in blood pressure by enhancing the sympathetic tone, increasing arterial stiffness, endothelial dysfunction, rising salt sensitivity, and increasing the Renin-angiotensin-aldosterone system (RAAS) [58, 59]. The RAAS increases by the decrease in eGFR (estimated Glomerular Filtration Rate), which leads to salt and water retention [60]. Endothelial dysfunction is characteristic of advanced CKD (30eGFR < ml/min / 1.73 m2), and its association with hypertension has been well established [61].

According to the results of this study, the risk of uncontrolled hypertension in non-cardiovascular patients was 1.5 times higher than in patients with cardiovascular disease. In the Almalki study in Saudi Arabia, uncontrolled hypertension was more common in subjects without a history of cardiac disease [35]. In the Aberhe et al. (2020) study, 86.7% of participants with uncontrolled hypertension had no history of cardiac disease [25]. However, in the study by Cherfan et al. (2020), a history of cardiovascular disease increased the prevalence of uncontrolled hypertension [34]. The observed difference in these results may be related to the difference in the method of analysis of the mentioned studies and the study group.

In this study, the prevalence of uncontrolled hypertension in smokers was 60.7%. Also, based on the multiple regression model, smoking in older adults and middle-aged participants did not have a significant relationship with uncontrolled hypertension, which is consistent with Masilela’s study (2020) [39]. Contrary to the present results, in a previous study, non-smokers had higher uncontrolled hypertension [34]. This inconsistency may be due to the difference in the age range in the mentioned study (age group of over 18) with this study (age group of 50–74). Some studies have shown that smoking has a negative effect on blood pressure control [62, 63]. For example, Cavusoglu et al. (2004) showed that smoking could cause direct endothelial damage, leading to endothelial dysfunction and endothelium-dependent coronary artery dilation [64]. In addition, smoking causes significant adverse outcomes in hemodynamics that affect small and large arteries [65] and damage the endothelium [66]. However, some other studies have reported no association between smoking and hypertension [67–69]. Even according to a study in the UK, hypertension does not decrease by quitting smoking [70].

According to the results of this study, the level of education was not related to uncontrolled hypertension. In a study, results showed that no significant relationship was observed between uncontrolled hypertension and education in South Africa [39]. Moreover, a systematic review of older adults in Africa showed that educational attainment was mostly not associated with hypertension [14]. However, in another study, uncontrolled hypertension was more common in participants with undergraduate education than in those with higher and postgraduate education in France [34]. These inconsistencies may be due to differences in the education classification between the above studies, which had been done according to successful years of study. In addition, the level of education of most participants in this study was less than a high school diploma, while in the above study, 39.8% of participants had postgraduate education. Although the prevalence of hypertension has been shown to increase with increasing illiteracy in previous study Since in low-income countries, those with higher education tend to be overweight or obese [71, 72], it may have been expected that higher education would predict hypertension if the relationships were linear or unconfounded [71]. Also, according to Azeez I. A et al. (2020) study, the prevalence of uncontrolled systolic blood pressure was higher in those with tertiary education than in those who were illiterate or had primary or secondary education. The prevalence of uncontrolled diastolic hypertension was higher in illiterate people than in those with primary, secondary, or tertiary education [73]. Individuals with lower education have more uncontrolled hypertension due to insufficient knowledge of the importance of hypertension and the necessary treatment and follow-up.

This study was one of the largest population-based studies in Iran. Other strengths of this research are an acceptable participation rate (85%), accurate implementation, and daily monitoring of the data collection process. The present study includes some limitations like the unavailability of information such as the amount of physical activity, the type of diet consumed, and the possible consumption of herbal teas and medicines for uncontrolled hypertension in middle-aged and older adults.

## Conclusion

This study showed a high prevalence of uncontrolled hypertension among middle-aged and older adults. Variables such as male gender, diabetes, and CKD also increased the risks of developing uncontrolled hypertension. Therefore, the health system authorities are expected to take the necessary measures to formulate and implement immediate actions for preventing or controlling hypertension.

## Data Availability

The datasets used and/or analyzed during the current study are available from the corresponding author on reasonable request.

## Abbreviations

ShECS: Shahroud Eye Cohort Study
CKD: Chronic kidney disease
OR: Odds Ratio
CI: Confidence Interval
WHO: World health organization
JNC 7: Joint National Committee 7
ACC: The American College of Cardiology
AHA: American Heart Association
NCD: Non-Communicable Diseases
SBP: Systolic Blood Pressure
DBP: Diastolic Blood Pressure
PCA: principal component analysis
BMI: Body Mass Index
GFR: Glomerular Filtration Rate
OTC: Over the Counter
